# Supplementary figures and images for: Time-resolved mitochondrial screen identifies regulatory components of oxidative metabolism
Source: EMBO Rep. 2025 Apr 29;26(12):3045–74. doi: 10.1038/s44319-025-00459-9 (PMC12187934; doi:10.1038/s44319-025-00459-9)

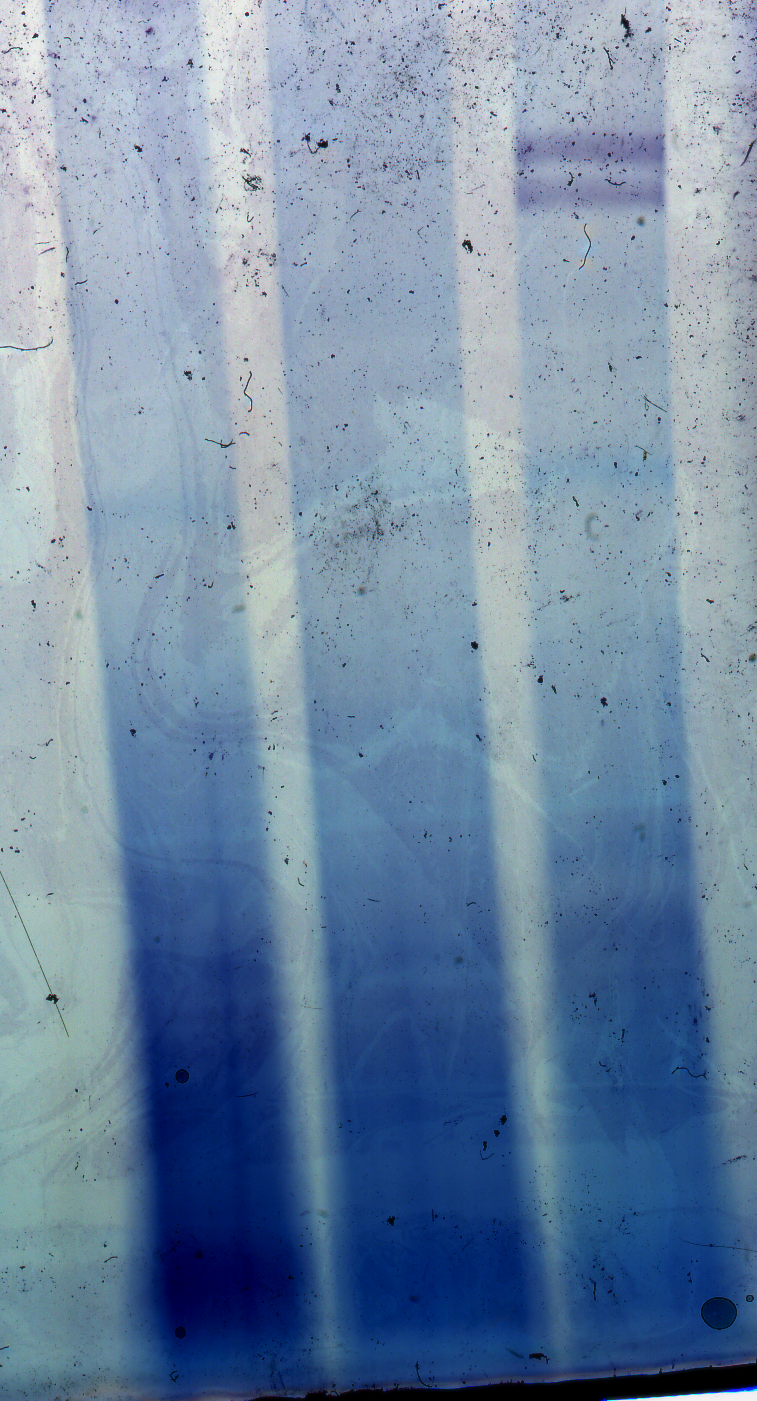

Supplement: Supplementary file 9 — Source data Fig. 4 [file 44319_2025_459_MOESM9_ESM.zip › Figure 4/4E/Sara_IGA_1.tif]

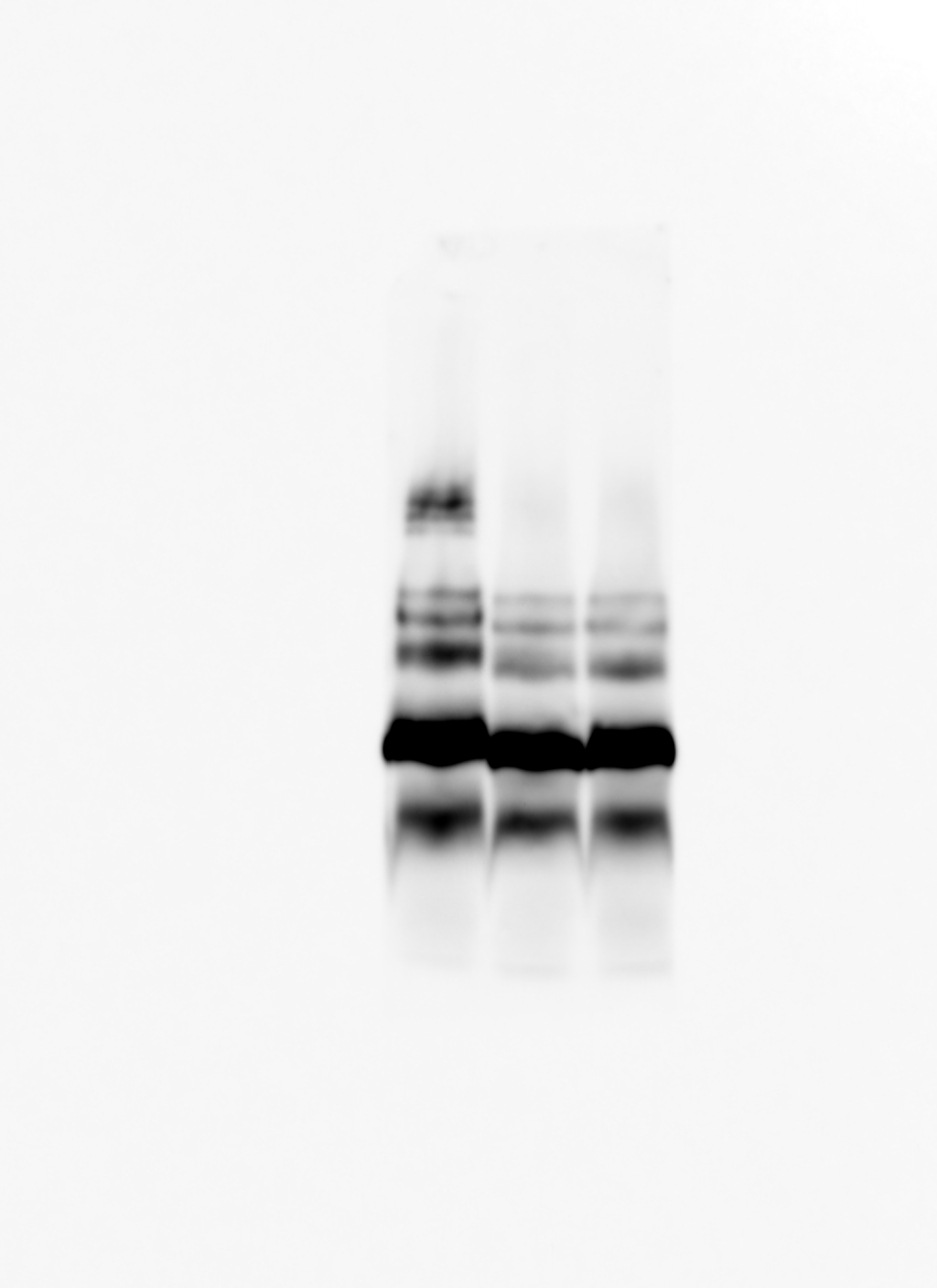

Supplement: Supplementary file 9 — Source data Fig. 4 [file 44319_2025_459_MOESM9_ESM.zip › Figure 4/4F/CIV (MT-CO1) R6,R3,V2 saturadaPAPER.tif]

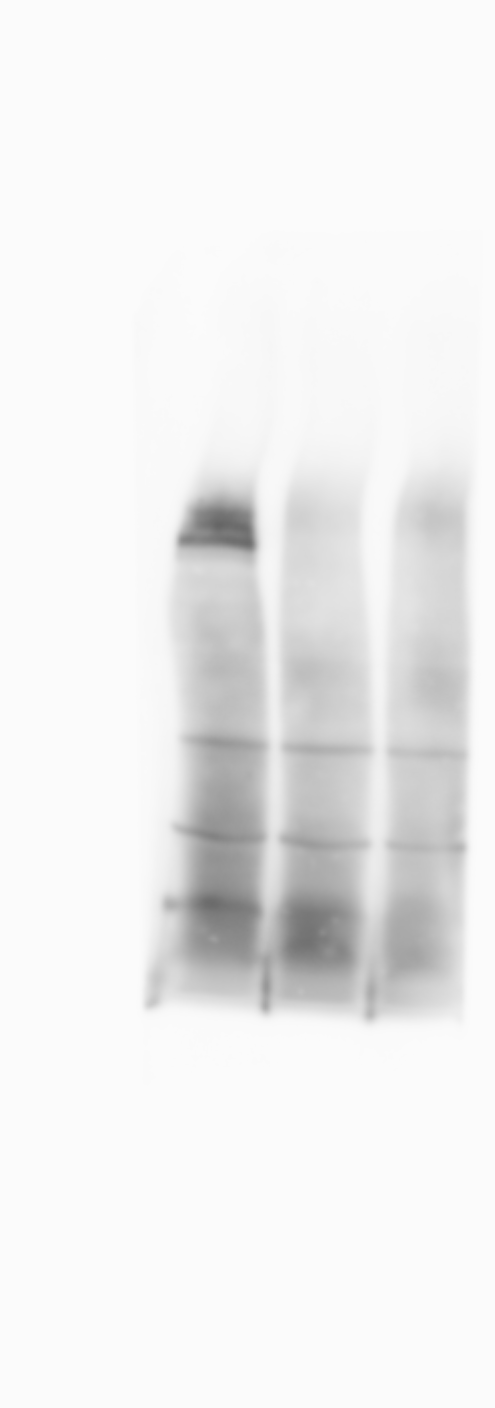

Supplement: Supplementary file 9 — Source data Fig. 4 [file 44319_2025_459_MOESM9_ESM.zip › Figure 4/4F/CI (NDUFA9) V2,R3,R6.tif]

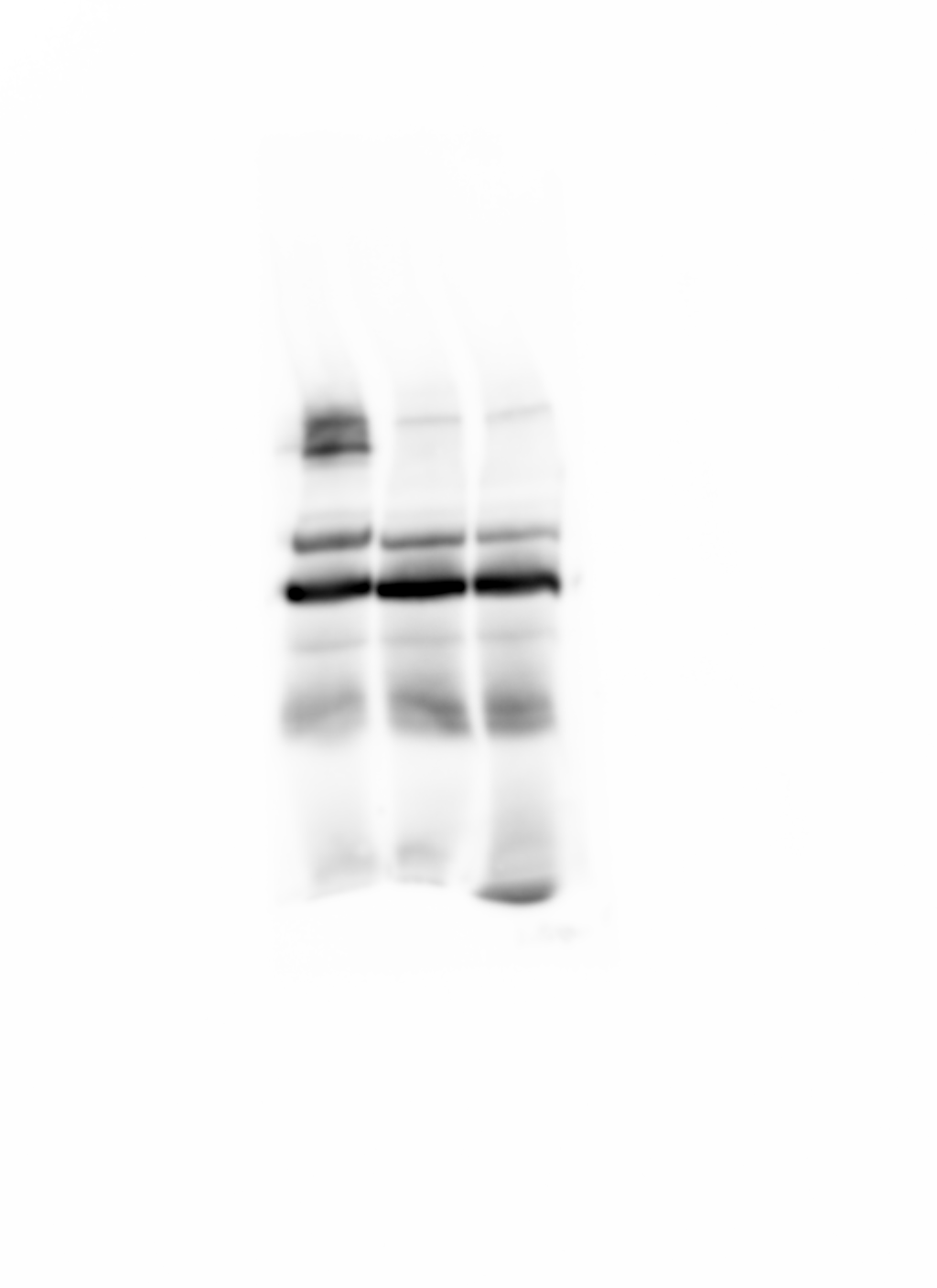

Supplement: Supplementary file 9 — Source data Fig. 4 [file 44319_2025_459_MOESM9_ESM.zip › Figure 4/4F/CIII (UQCRQ) V2,R3,R6PAPER.tif]

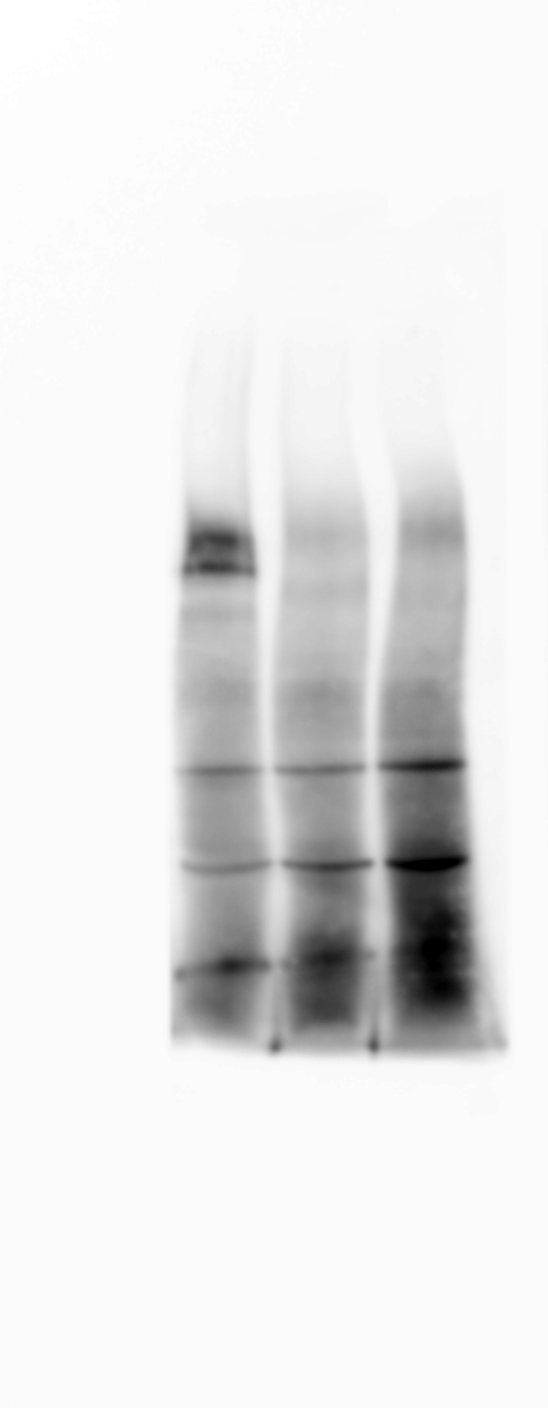

Supplement: Supplementary file 10 — Source data Fig. 5 [file 44319_2025_459_MOESM10_ESM.zip › Figure 5/5E/CI (NDUFA9) V2,R3,R3CoQPAPER.tif]

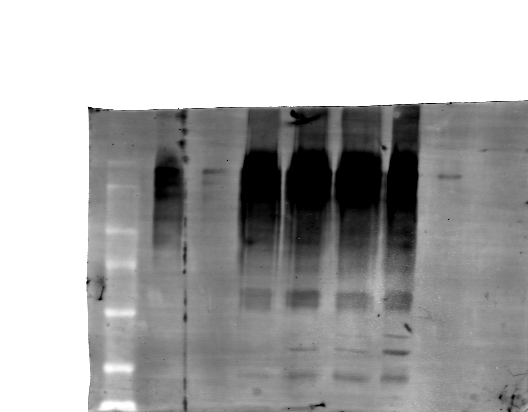

Supplement: Supplementary file 11 — Source data Fig. 6 [file 44319_2025_459_MOESM11_ESM.zip › Figure 6/6O/PDH .tif]

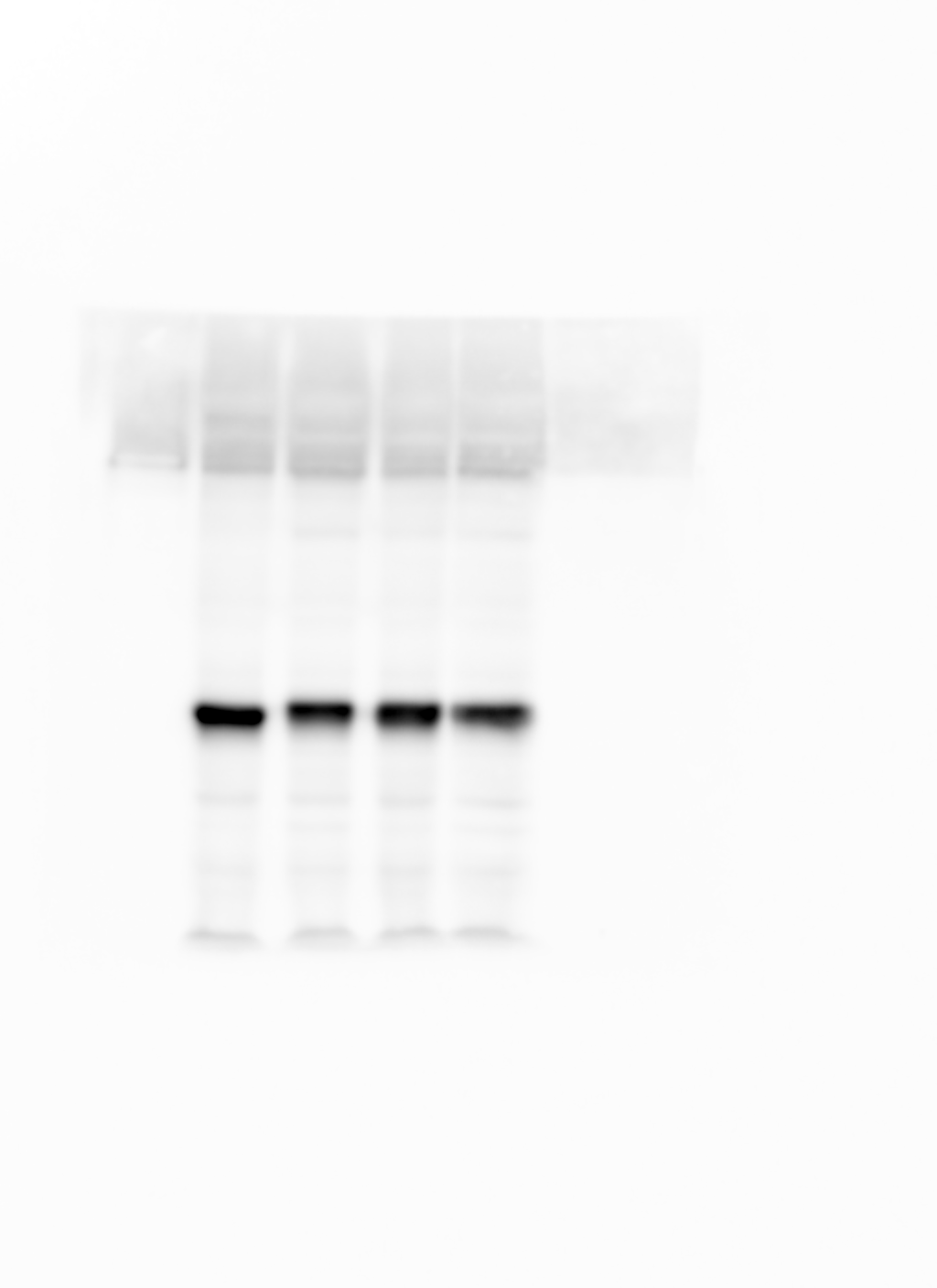

Supplement: Supplementary file 11 — Source data Fig. 6 [file 44319_2025_459_MOESM11_ESM.zip › Figure 6/6O/IP PDH lnputPDH.tif]

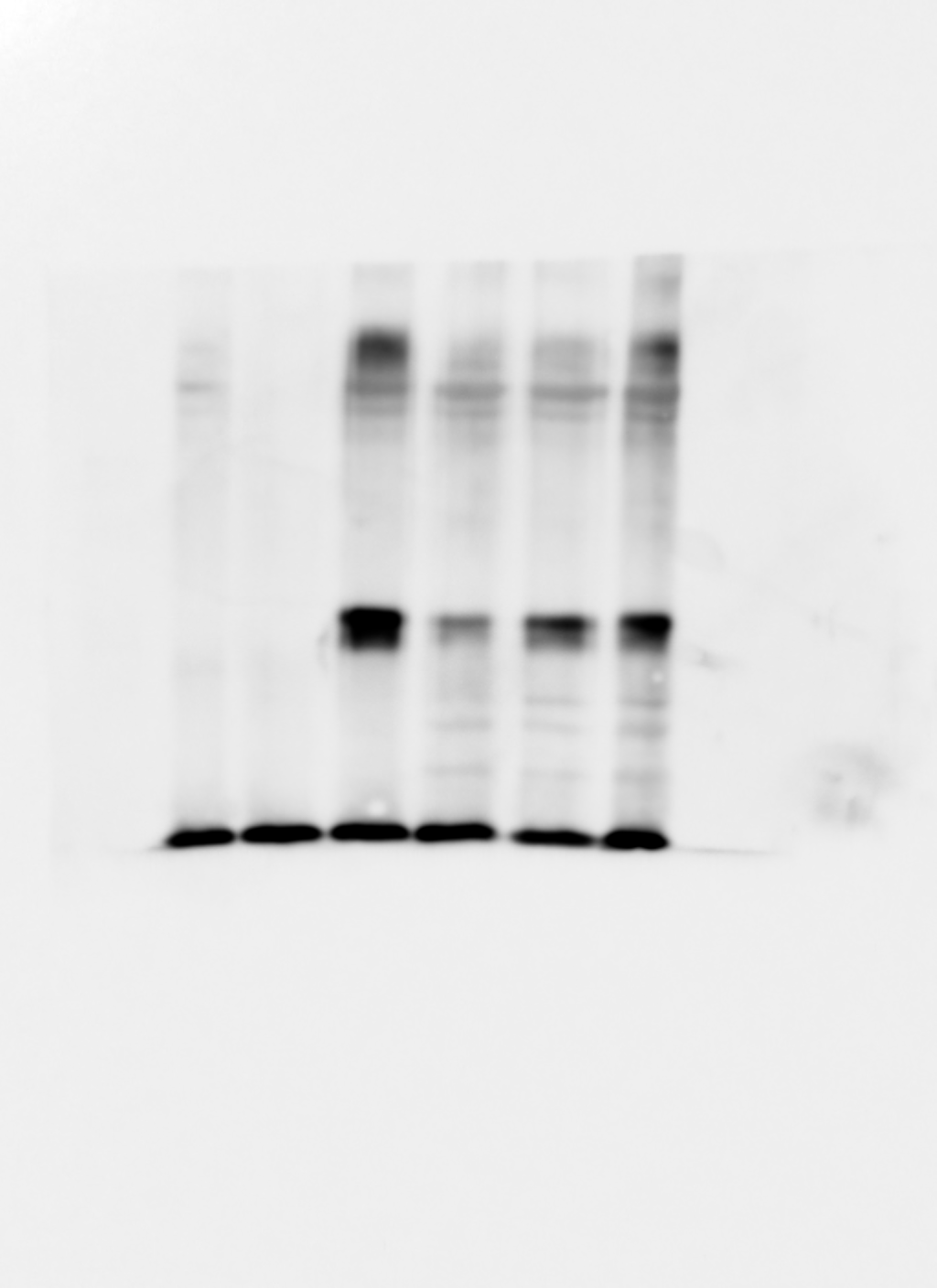

Supplement: Supplementary file 11 — Source data Fig. 6 [file 44319_2025_459_MOESM11_ESM.zip › Figure 6/6O/IP PDH lipoic .tif]

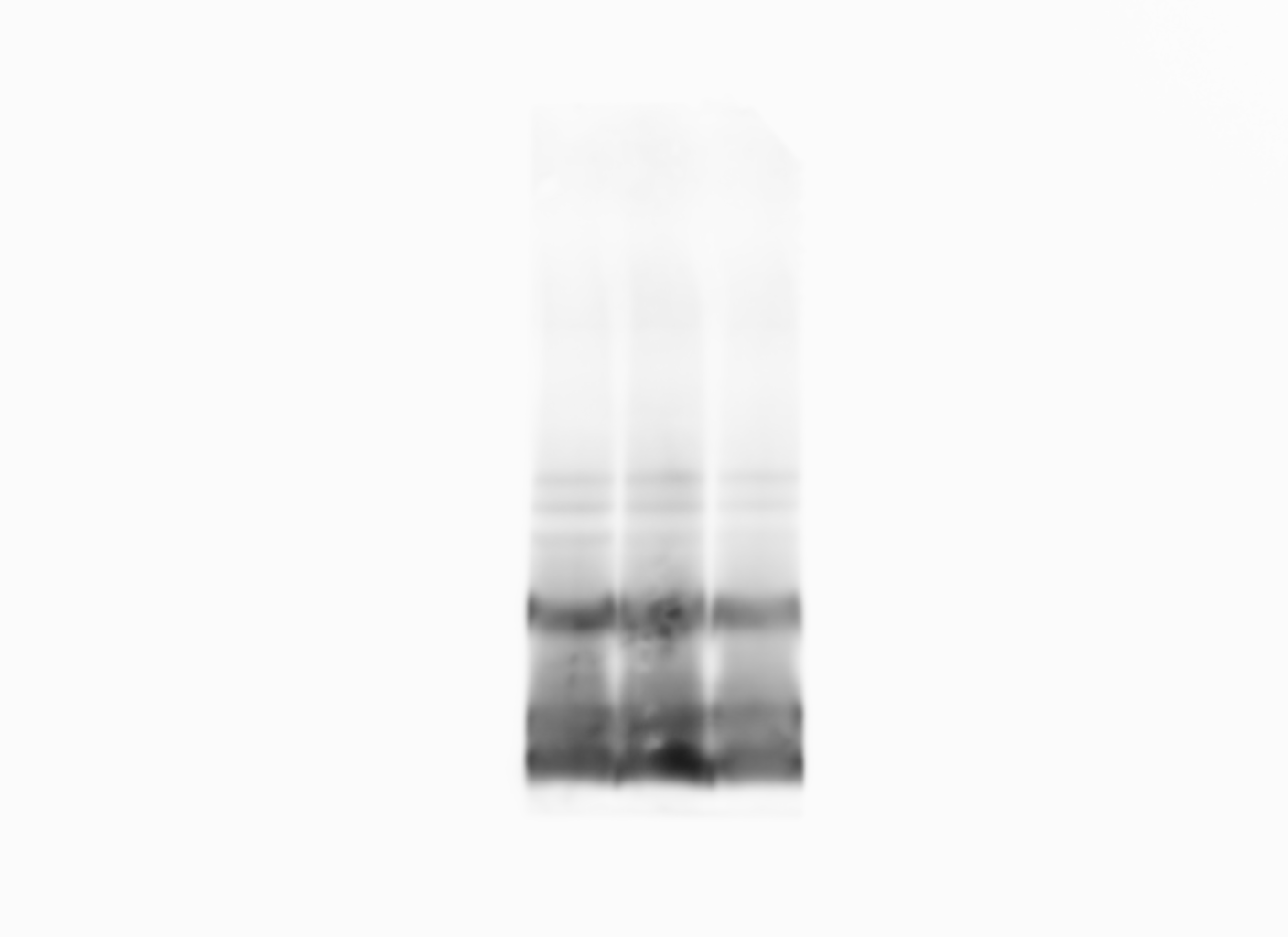

Supplement: Supplementary file 11 — Source data Fig. 6 [file 44319_2025_459_MOESM11_ESM.zip › Figure 6/6D/CII .tif]

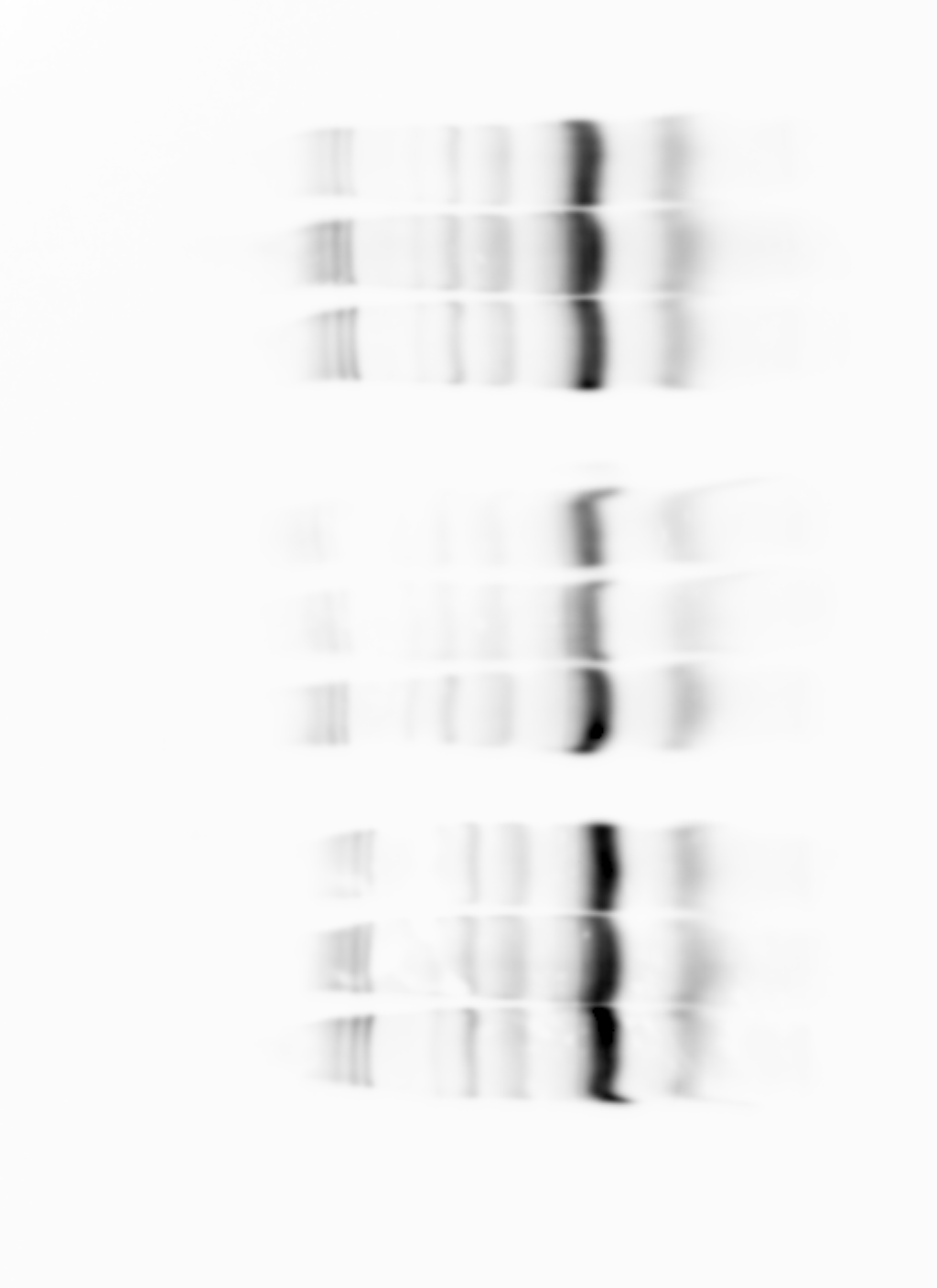

Supplement: Supplementary file 11 — Source data Fig. 6 [file 44319_2025_459_MOESM11_ESM.zip › Figure 6/6D/MTCO1 .tif]

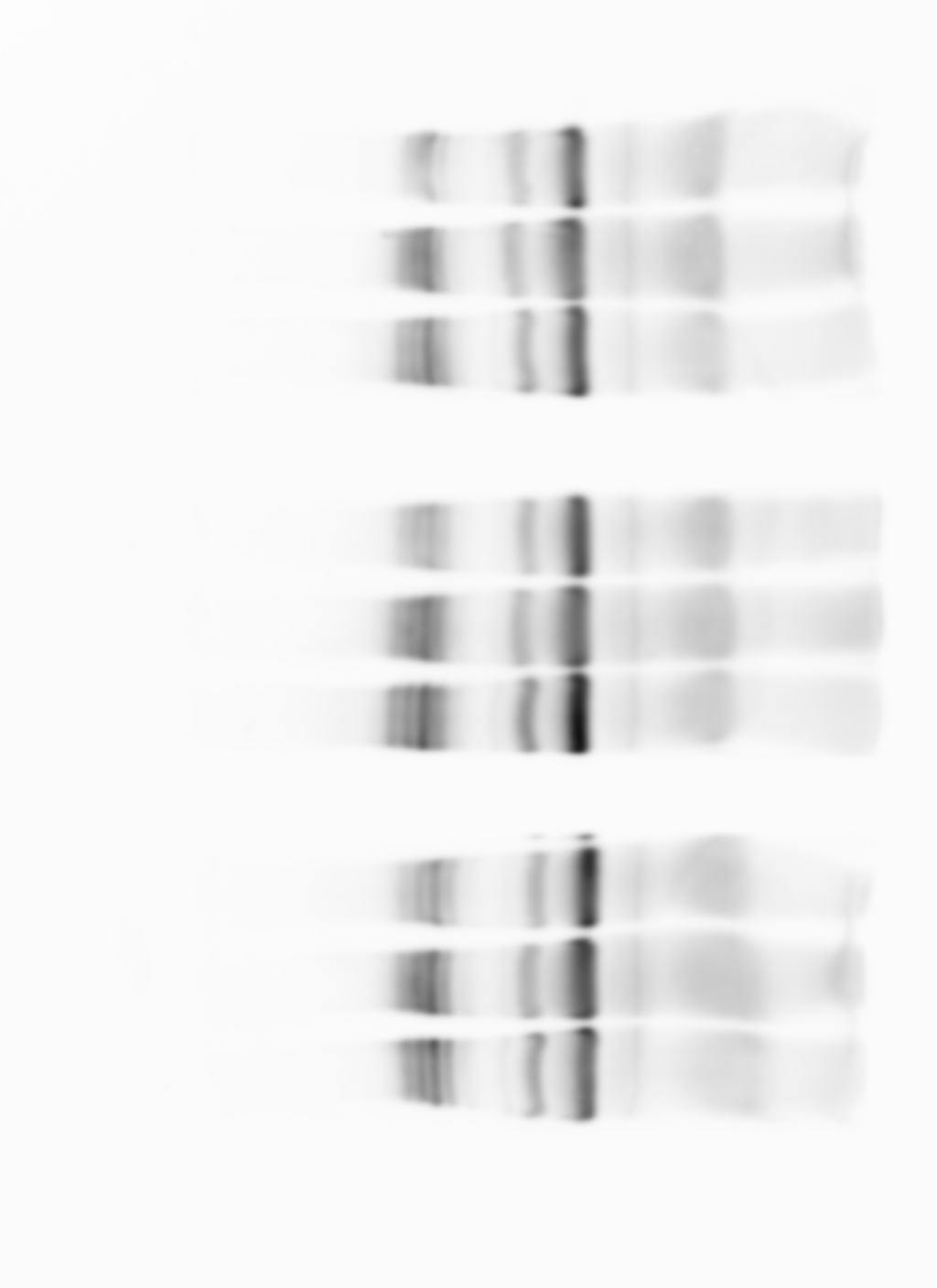

Supplement: Supplementary file 11 — Source data Fig. 6 [file 44319_2025_459_MOESM11_ESM.zip › Figure 6/6D/UQCRQ .tif]

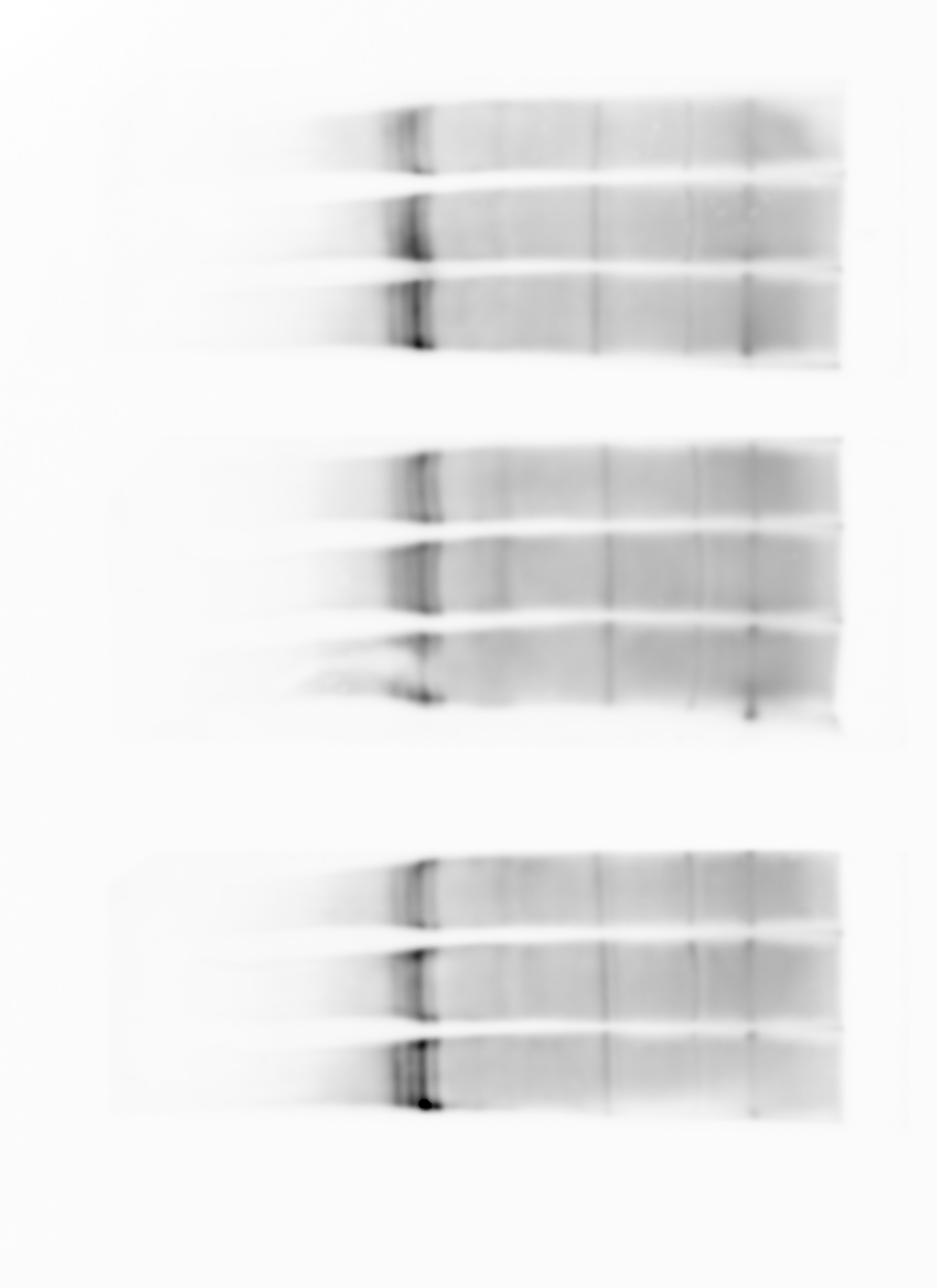

Supplement: Supplementary file 11 — Source data Fig. 6 [file 44319_2025_459_MOESM11_ESM.zip › Figure 6/6D/NDUF9 .tif]

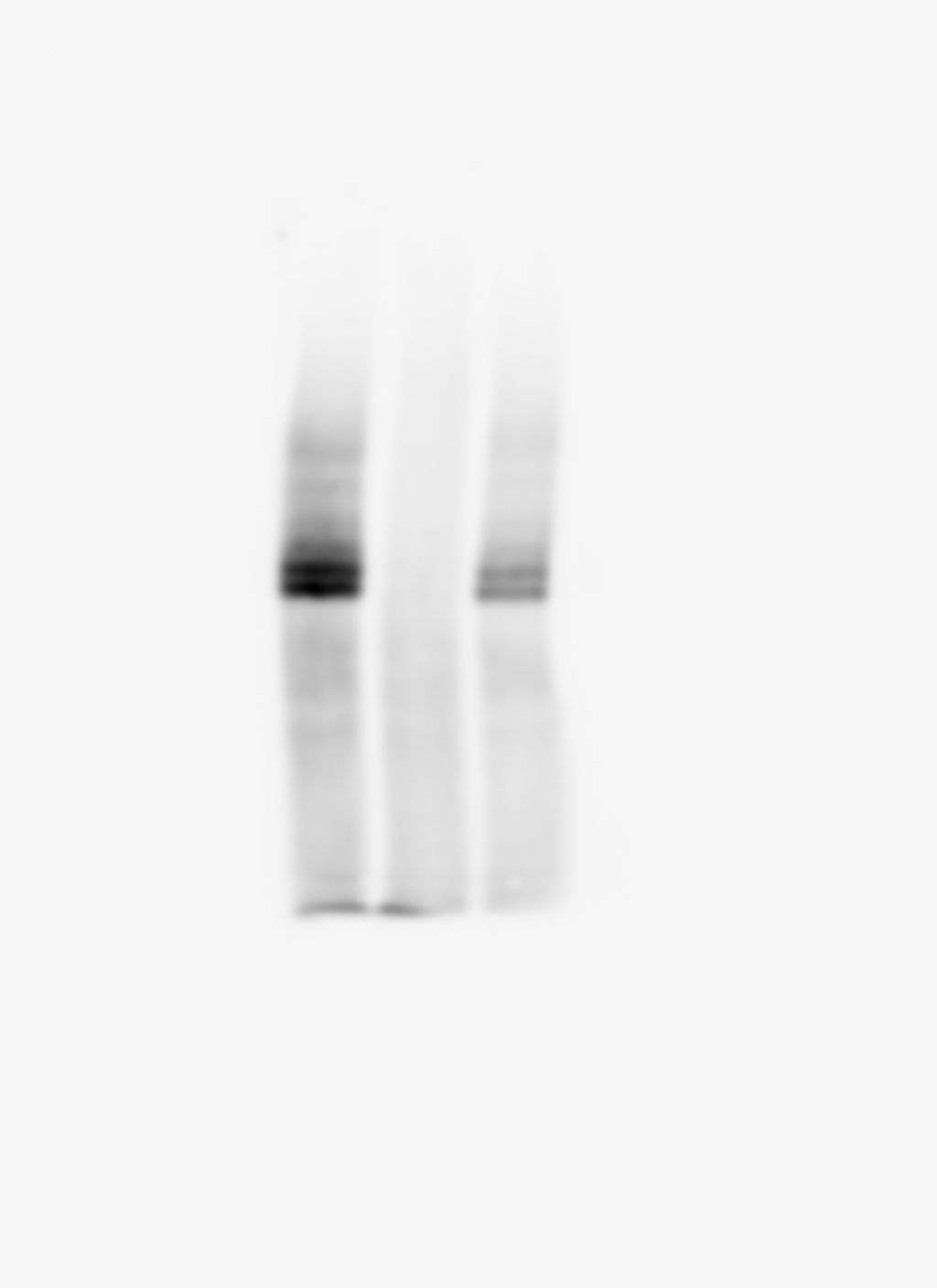

Supplement: Supplementary file 12 — Figure Source Data All EV [file 44319_2025_459_MOESM12_ESM.zip › Extended view 4/4D/4D.tif]

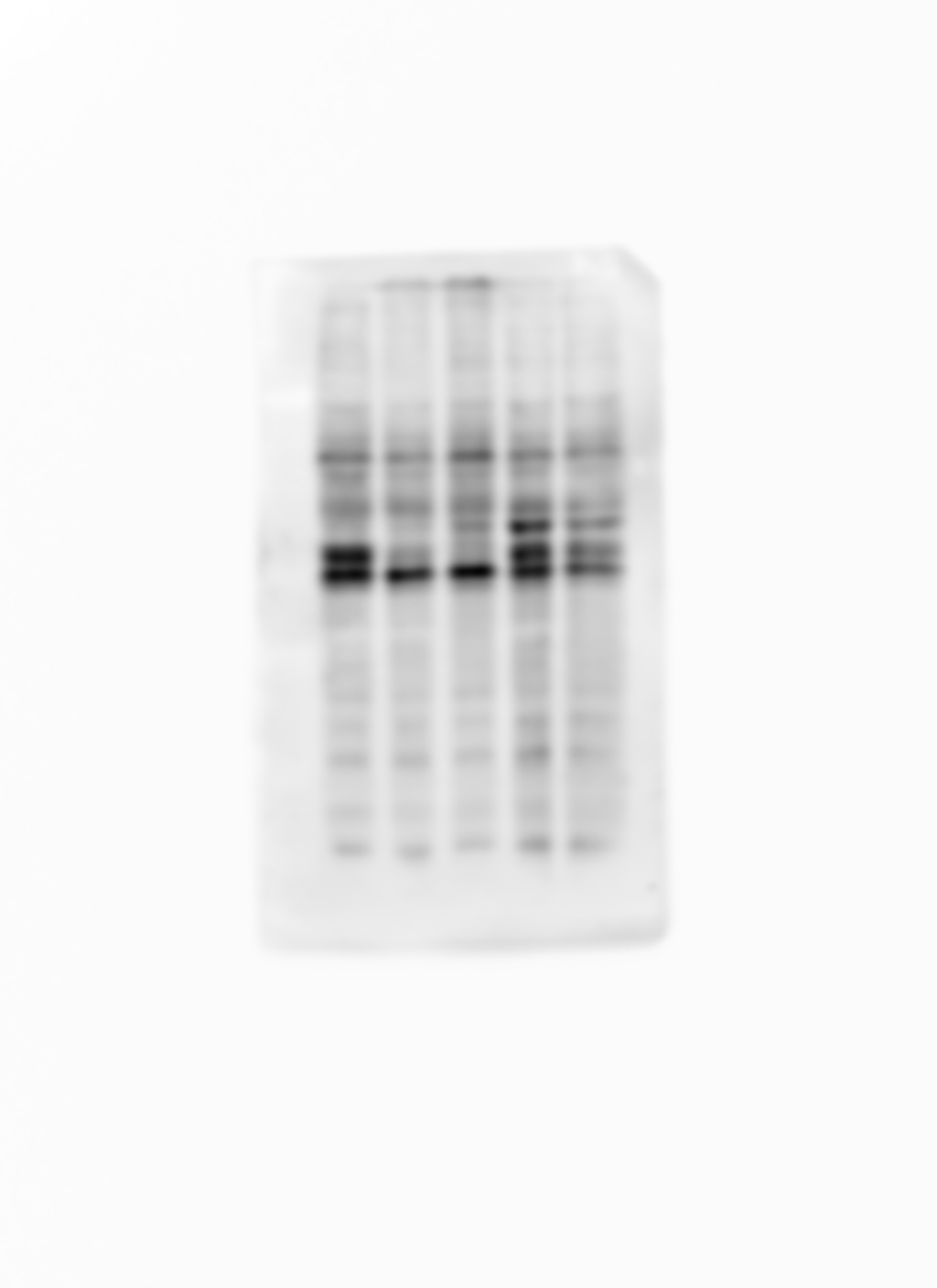

Supplement: Supplementary file 12 — Figure Source Data All EV [file 44319_2025_459_MOESM12_ESM.zip › Extended view 4/4A/RTN4IP1 (WT, KO, KO, SOBRE, SOBRE).tif]

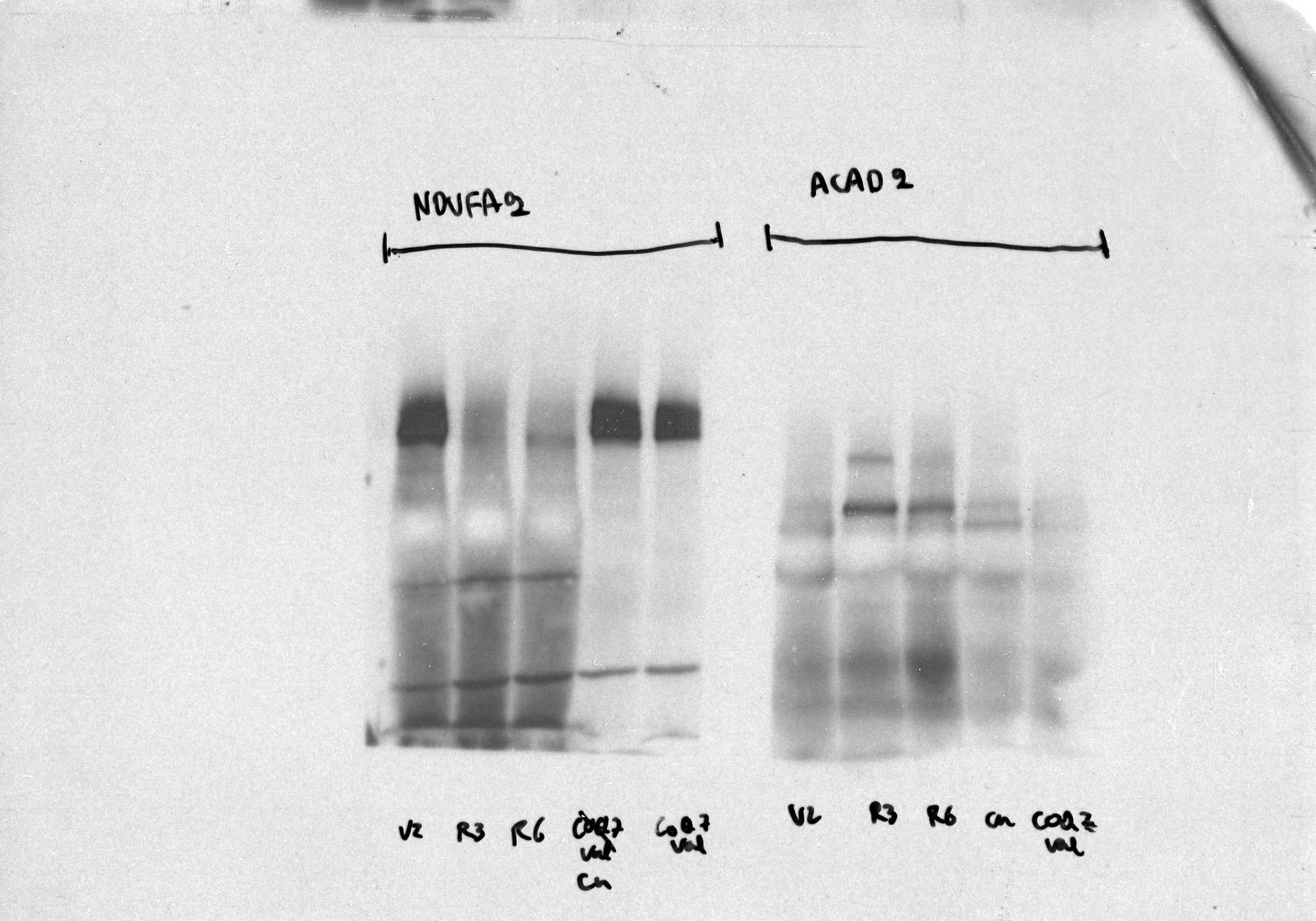

Supplement: Supplementary file 12 — Figure Source Data All EV [file 44319_2025_459_MOESM12_ESM.zip › Extended view 5/5H/BN mutants CoQ.jpg]

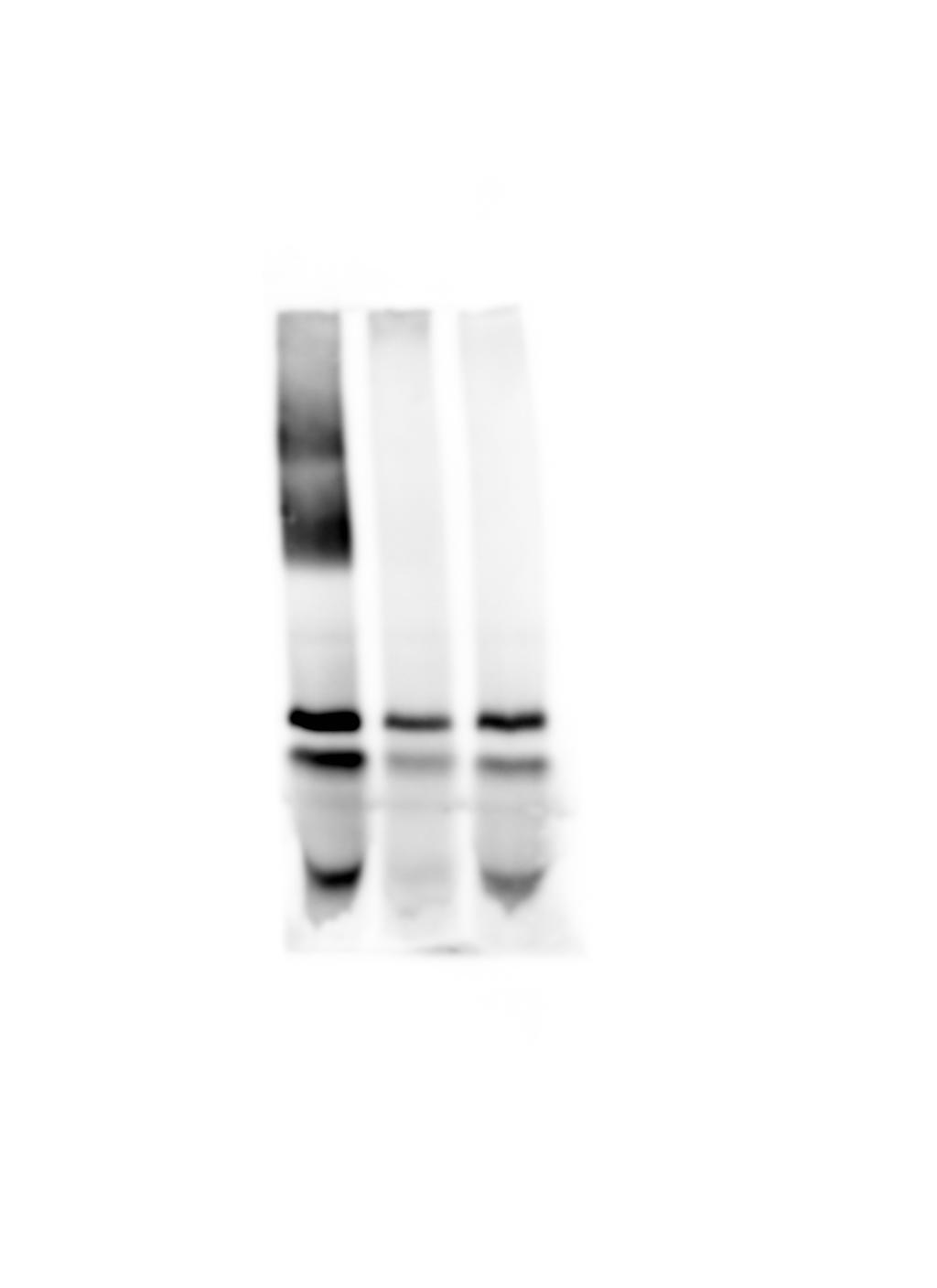

Supplement: Supplementary file 12 — Figure Source Data All EV [file 44319_2025_459_MOESM12_ESM.zip › Extended view 5/5E/BN NDUFV1 .jpg]

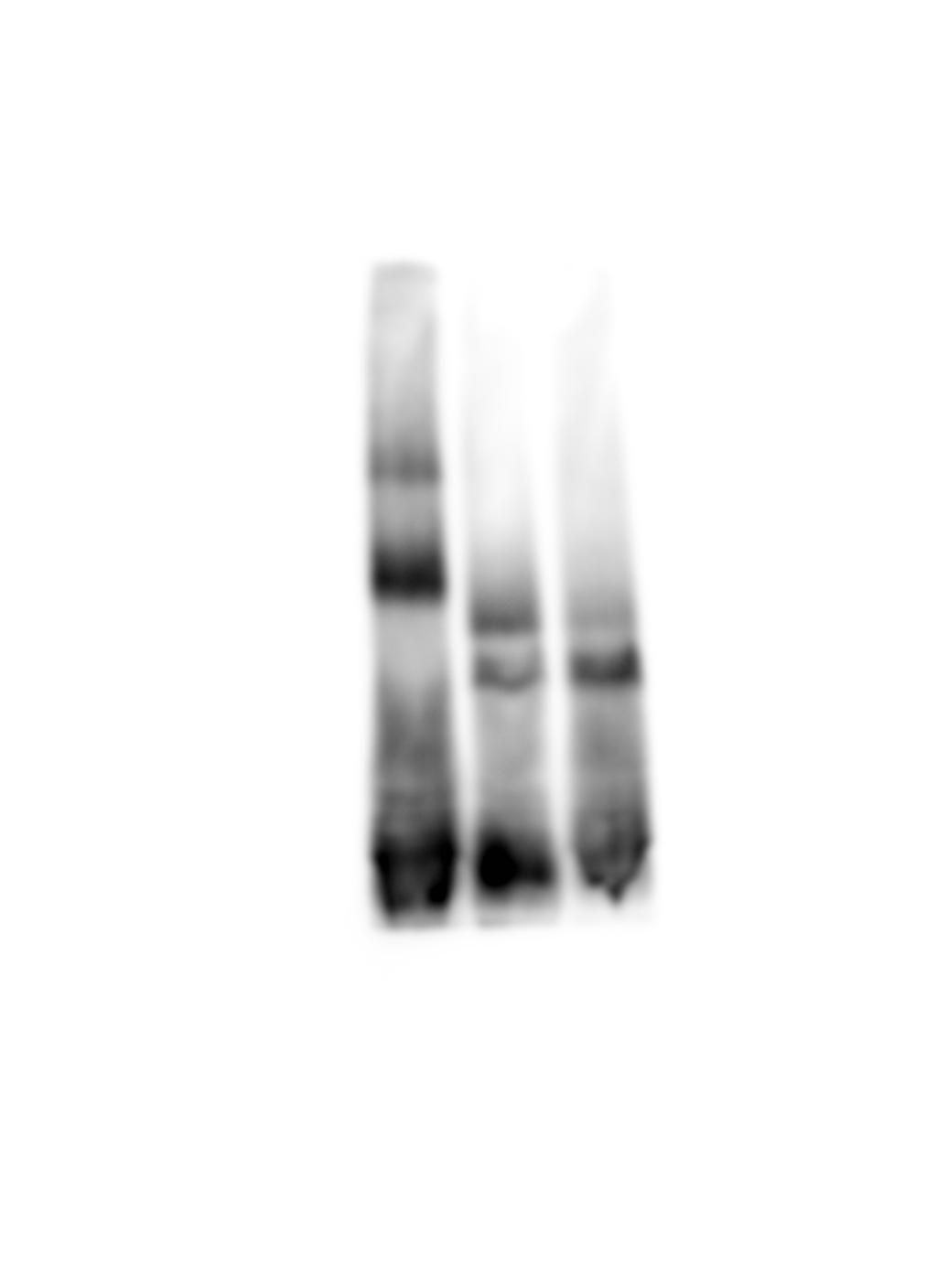

Supplement: Supplementary file 12 — Figure Source Data All EV [file 44319_2025_459_MOESM12_ESM.zip › Extended view 5/5E/BN NDUFS2.jpg]

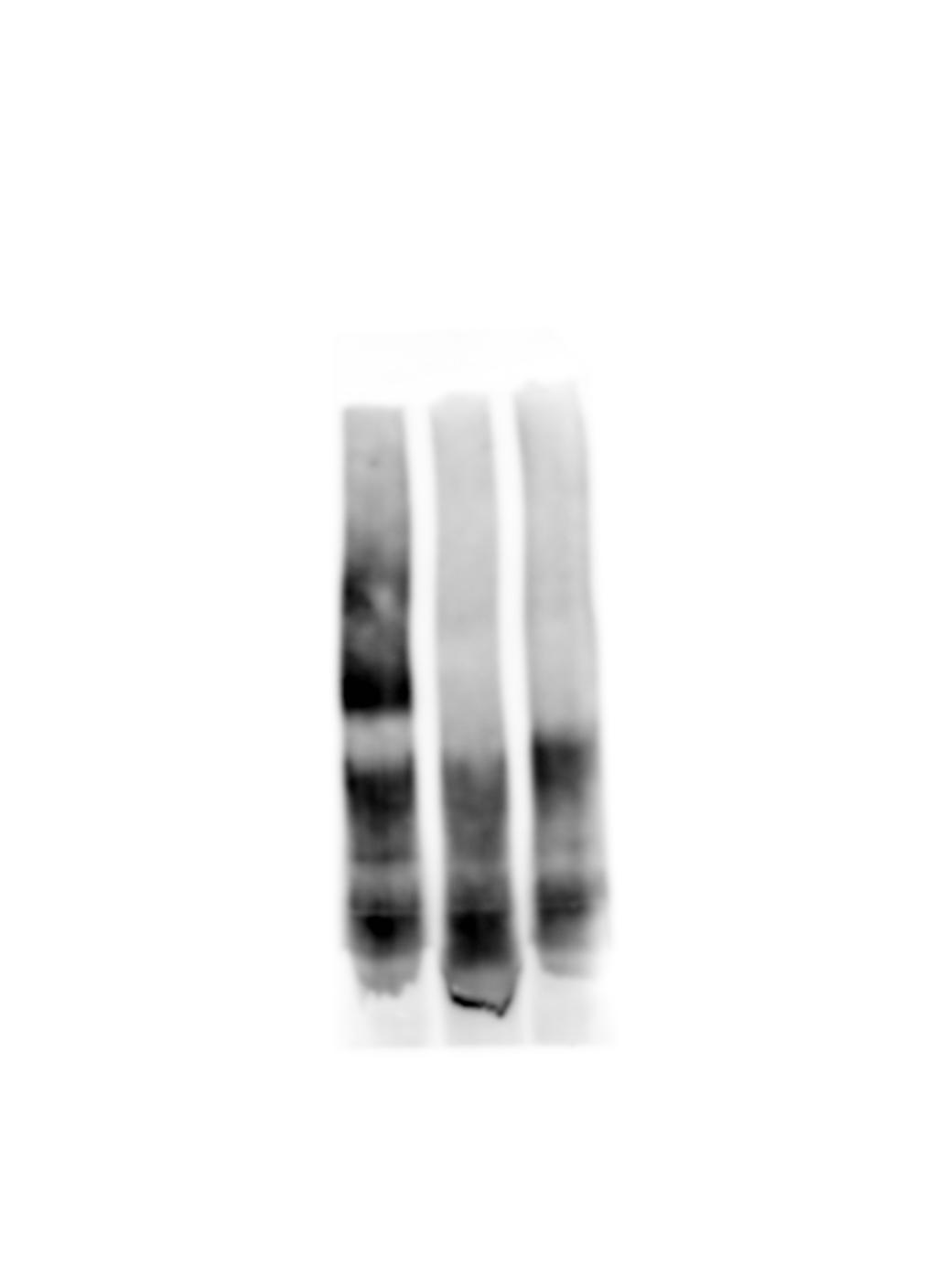

Supplement: Supplementary file 12 — Figure Source Data All EV [file 44319_2025_459_MOESM12_ESM.zip › Extended view 5/5E/BN NDUFB7 .jpg]

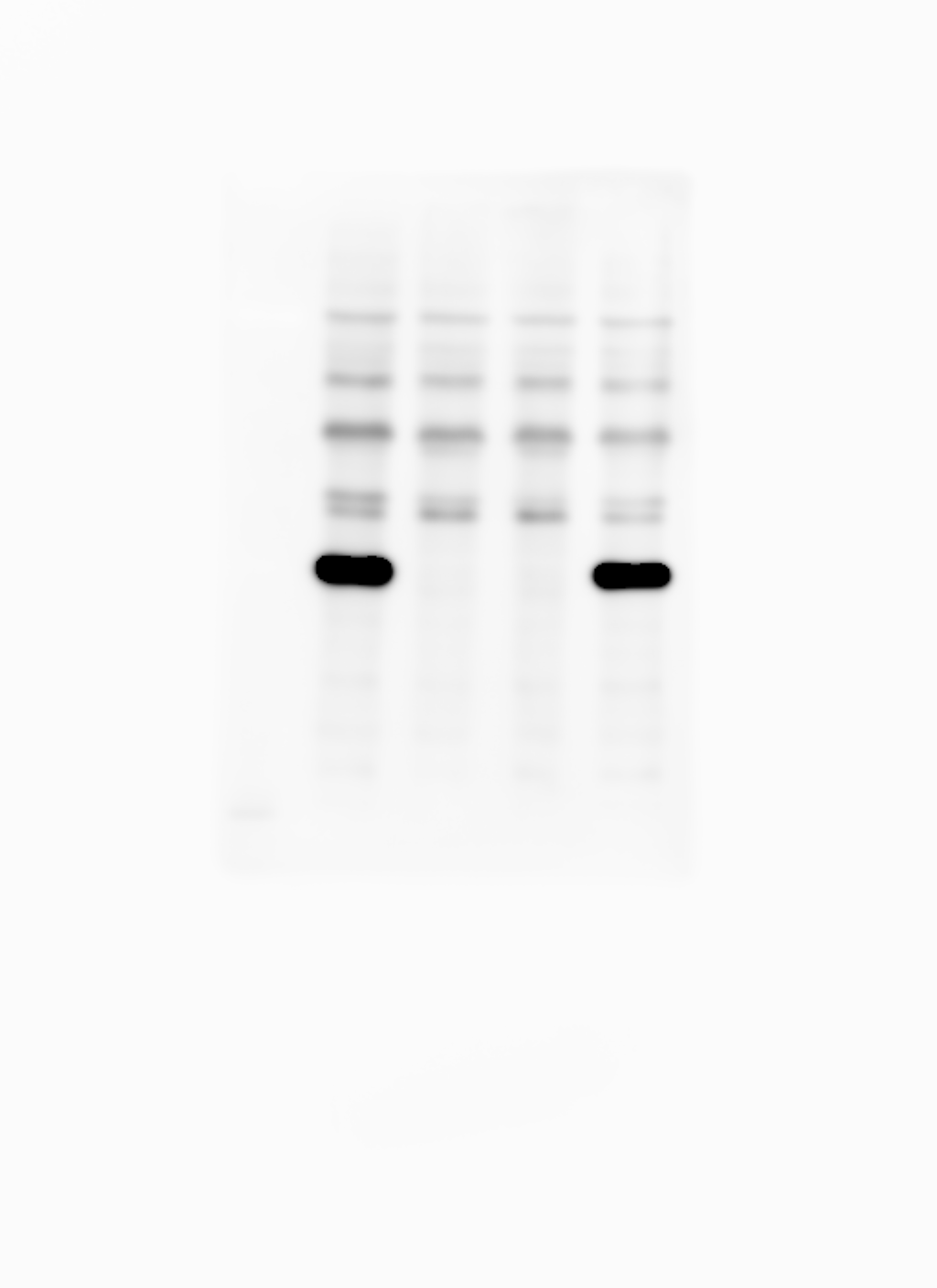

Supplement: Supplementary file 12 — Figure Source Data All EV [file 44319_2025_459_MOESM12_ESM.zip › Extended view 6/6A/ECHS1 (WT, E11, E13, SOBREEXP).tif]

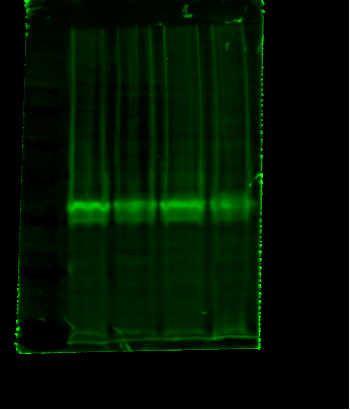

Supplement: Supplementary file 12 — Figure Source Data All EV [file 44319_2025_459_MOESM12_ESM.zip › Extended view 6/6O/WB pdhPAPER.tif]

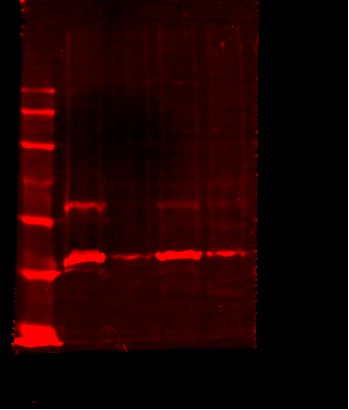

Supplement: Supplementary file 12 — Figure Source Data All EV [file 44319_2025_459_MOESM12_ESM.zip › Extended view 6/6O/WB Lipoico ECHSPAPER.tif]
