# Supplementary material for: Time-resolved mitochondrial screen identifies regulatory components of oxidative metabolism
Source: EMBO Rep. 2025 Apr 29;26(12):3045–74. doi: 10.1038/s44319-025-00459-9 (PMC12187934; doi:10.1038/s44319-025-00459-9)
Supplement: Supplementary file 13 — Expanded View Figures [file 44319_2025_459_MOESM13_ESM.pdf]

# Expanded View Figures

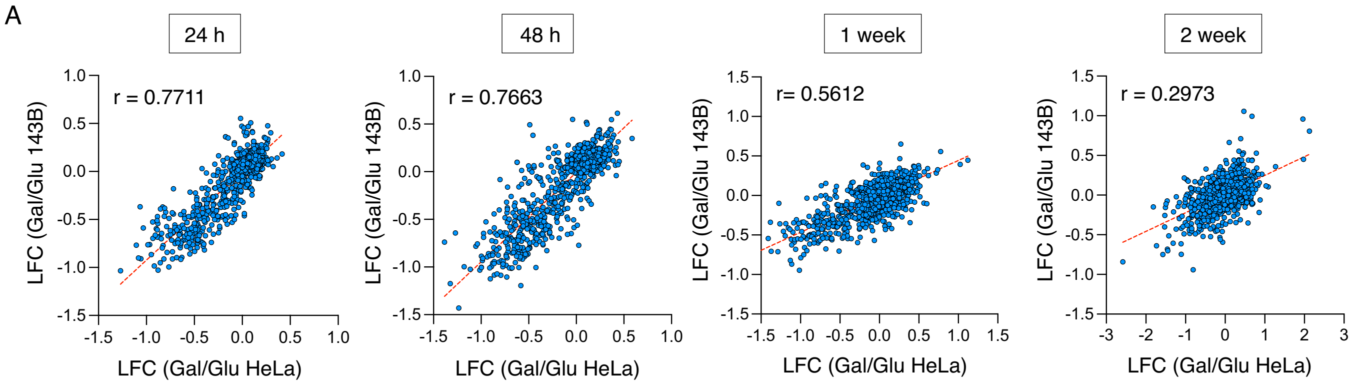


**Figure EV1. Correlation between HeLa and 143B screening results.**

**(A)** Correlative analysis of the logarithmic fold change in the galactose/glucose ratio between HeLa and 143B cells at the indicated time points. Source data are available online for this figure.

A

Dynamics and surveillance

| Severe Phe. | Dynamics and surveillance |
|---|---|
| 24 hours | ATP5ME CYCS |
| 48 hours | CYCS ATP5ME |
| 1 week | |
| 2 weeks | |

B

Signaling

| Severe Phe. | Signaling |
|---|---|
| 24 hours | |
| 48 hours | |
| 1 week | |
| 2 weeks | SLC25A5 |

D

Transport

| Severe Phe. | Protein import | Small molecule transport |
|---|---|---|
| 24 hours | UQCRC1 | |
| 48 hours | UQCRC1 | |
| 1 week | UQCRC1 | SLC25A3 SLC25A51 SLC25A19 SLC25A1 |
| 2 weeks | UQCRC1 TOMM22 TIMM21 | SFXN4 SLC25A51 SLC25A1 MPC2 SLC25A28 SLC25A37 SLC25A5 |

C

MitoCD

| Severe Phe. | mtDNA maintenance | mtRNA metabolism | mtRNA translation |
|---|---|---|---|
| 24 hours | POLRMT | PRORP POLRMT TRMT10C LRPPRC HSD17B10 | MRPL15 MRPS30 MRPS33 MRPS25 MRPL33 MRPL41 LRPPRC QRSL1 MRPS12 MRPL46 MRPS22 MRPL18 AURKAIP1 MRPL57 NARS2 TUFM YARS2 MRPL4 MRPL23 MARS2 MRPL44 VARS2 MRPS18A |
| 48 hours | TFAM TWNK POLRMT | DDX28 TFAM METTL17 MTPAP LRPPRC FASTKD5 RPUSD4 MRPL12 POLRMT PRORP HSD17B10 | MRPS30 MRPL20 NARS2 MRPL34 MRPL37 MRPL15 QRSL1 TUFM YARS2 MRPL17 MRPL46 MRPL40 MRPS22 LARS2 MRPS18A PTCD3 MRPL12 MRPS33 MRPL4 AARS2 MARS2 VARS2 MRPS6 MRPL23 MRPS23 MRPL57 MRPL38 SARS2 MRPL41 DDX28 MRPL33 PARS2 MRPL44 LRPPRC MRPS12 METTL17 |
| 1 week | | FASTKD5 METTL17 PDE12 GRSF1 MTO1 RMND1 MRM2 | MTRF1 GRSF1 MTIF3 COA3 RMND1 METTL17 GFM2 TACO1 MRM2 |
| 2 weeks | POLDIP2 | PTCD2 MTO1 PDE12 MRM2 GTPBP3 | HEMK1 MRM2 TIMM21 TACO1 |

E

Metabolism

| Severe Phe. | Carbohydrate | Lipid | Protein | Nucleotide | Vitamin | Detoxification | Electron carriers | Metal and cofactor |
|---|---|---|---|---|---|---|---|---|
| 24 hours | | HSD17B10 | HSD17B10 | | | | CYC1 CYCS | ISCA1 CYC1 CYCS COX17 COQ4 NDUFS1 NDUFV1 UQCRFS1 NDUFS2 ISCA2 NDUFV2 GLRX5 |
| 48 hours | DLST DLD SDHB OGDH | LIPT2 HSD17B10 LIPT1 | DLST HSD17B10 DLD | | FDX1 | SOD2 | CYCS CYC1 | COQ2 NDUFV1 CYC1 COX17 NDUFS2 ISCA2 GLRX5 CYCS SDHB NFU1 ISCA1 COQ4 NDUFV2 NDUFS8 FDX1 LIAS COX15 UQCRFS1 NDUFS1 |
| 1 week | SDHD OGDH DLD SDHC SDHB PDHB PDHA1 DLAT CS FH DLST SLC25A1 | MECR MCAT SLC25A1 OXSM LIAS LIPT2 ETFB FDX1 GCSH LIPT1 | ETFB GCSH DLST DLD | NME6 | SLC25A19 ETFB FDX1 MTO1 | SOD2 | ETFB CYC1 | SDHD SDHC NDUFS1 NDUFV2 LIAS BOLA3 GLRX5 SLC25A3 HCCS ISCA1 SLC25A51 PDSS2 UQCRFS1 COQ2 COQ5 COX17 SCO1 SDHB CYC1 NFU1 COQ6 NDUFS2 COX15 NUBPL FDX1 COQ7 |
| 2 weeks | SDHB PDHX PDHA1 PDPR DLD PDHB SDHD MDH2 SLC25A1 MPC2 DLAT OGDH CS | LIPT1 ETFA GCSH LIAS MECR ETFB MCAT OXSM LIPT2 ETFDH ECHS1 SLC25A1 HTD2 FDX1 | HIBCH ECHS1 GLUD1 GCSH ETFDH ETFB DLD ETFA | AK3 NME6 SLC25A5 | ETFDH FDX1 RFK GTPBP3 MTO1 ETFB ETFA SLC25A19 | SOD2 | CYC1 ETFDH ETFA ETFB | NUDT19 CYC1 SLC25A51 GLRX5 NDUFS2 COQ2 SLC25A28 SDHB SLC25A37 NFU1 SDHD LIAS FDX1 BOLA3 ETFDH COQ7 |

F

OXPHOS

| Severe Phe. | Core subunits | Assembly factors |
|---|---|---|
| 24 hours | COX7C CYC1 NDUFB2 COX5A NDUFC1 UQCRC1 NDUFB4 NDUFA1 NDUFV1 ATP5F1A NDUFB10 CYCS NDUFS2 UQCRB NDUFS1 NDUFB5 NDUFA8 UQCRFS1 NDUFB6 COX4I1 NDUFA2 NDUFS5 NDUFB11 UQCR10 NDUFA6 NDUFB9 NDUFB8 NDUFV2 NDUFA11 | COA6 NDUFAF5 COX16 NDUFAF4 BCS1L COX17 NDUFAF8 COA5 |
| 48 hours | NDUFB7 NDUFV1 NDUFB1 UQCRC1 NDUFB2 NDUFS1 NDUFA1 CYCS NDUFA3 NDUFA6 NDUFA8 UQCRFS1 NDUFC1 NDUFS5 COX5A SDHB UQCRB NDUFB10 COX4I1 NDUFB8 NDUFB4 NDUFS8 NDUFB11 ATP5ME NDUFS2 NDUFV2 NDUFB6 ATP5F1C NDUFA4 CYC1 NDUFA2 NDUFB9 NDUFA11 | NDUFAF3 COA6 COX17 NDUFAF8 COA5 NDUFAF4 DMAC1 BCS1L NDUFAF5 NDUFAF6 FOXRED1 COX15 COX16 |
| 1 week | COX4I1 HCCS UQCR11 NDUFV2 CYC1 NDUFA11 NDUFC1 SDHD UQCRC1 NDUFB10 NDUFA3 NDUFA8 NDUFB2 NDUFA1 SDHC COX6C NDUFS1 SDHB NDUFB8 NDUFA4 NDUFB11 NDUFA10 NDUFB5 NDUFA2 NDUFS5 NDUFB6 NDUFA6 NDUFS2 NDUFB7 NDUFB9 UQCRFS1 NDUFB4 | COA3 COX17 TACO1 NUBPL NDUFAF6 NDUFAF8 PET100 NDUFAF1 DMAC1 COX18 DMAC2 SDHAF1 COX16 ECSIT COA7 LYRM7 NDUFAF4 FOXRED1 COA6 SCO1 COX15 HIGD2A NDUFAF5 TIMMDC1 UQCC2 NDUFAF3 PET117 SURF1 |
| 2 weeks | NDUFB9 SDHD NDUFB4 UQCR11 NDUFB7 NDUFB8 NDUFS2 UQCRC1 NDUFB11 NDUFS5 NDUFA4 NDUFA11 NDUFA3 NDUFA10 NDUFB10 NDUFA1 SDHB CYC1 NDUFB2 NDUFB6 NDUFC1 | PET117 UQCC1 NDUFAF4 FOXRED1 UQCC2 SDHAF1 TIMMDC1 DMAC2 TIMM21 NDUFAF5 TACO1 CEP89 COX16 DMAC1 HIGD2A LYRM7 NDUFAF6 NDUFAF1 COX18 NDUFAF3 SURF1 |

 © The Author(s)

**Figure EV2. Genes with positive phenotype in the screening.**

Tables showing the genes that score positive with severe phenotype across different time points for the following functional groups: (**A**) Dynamics and surveillance, (**B**) Signaling, (**C**) Mitochondrial central dogma, (**D**) Transport, (**E**) Metabolism, and (**F**) OXPHOS. Source data are available online for this figure.

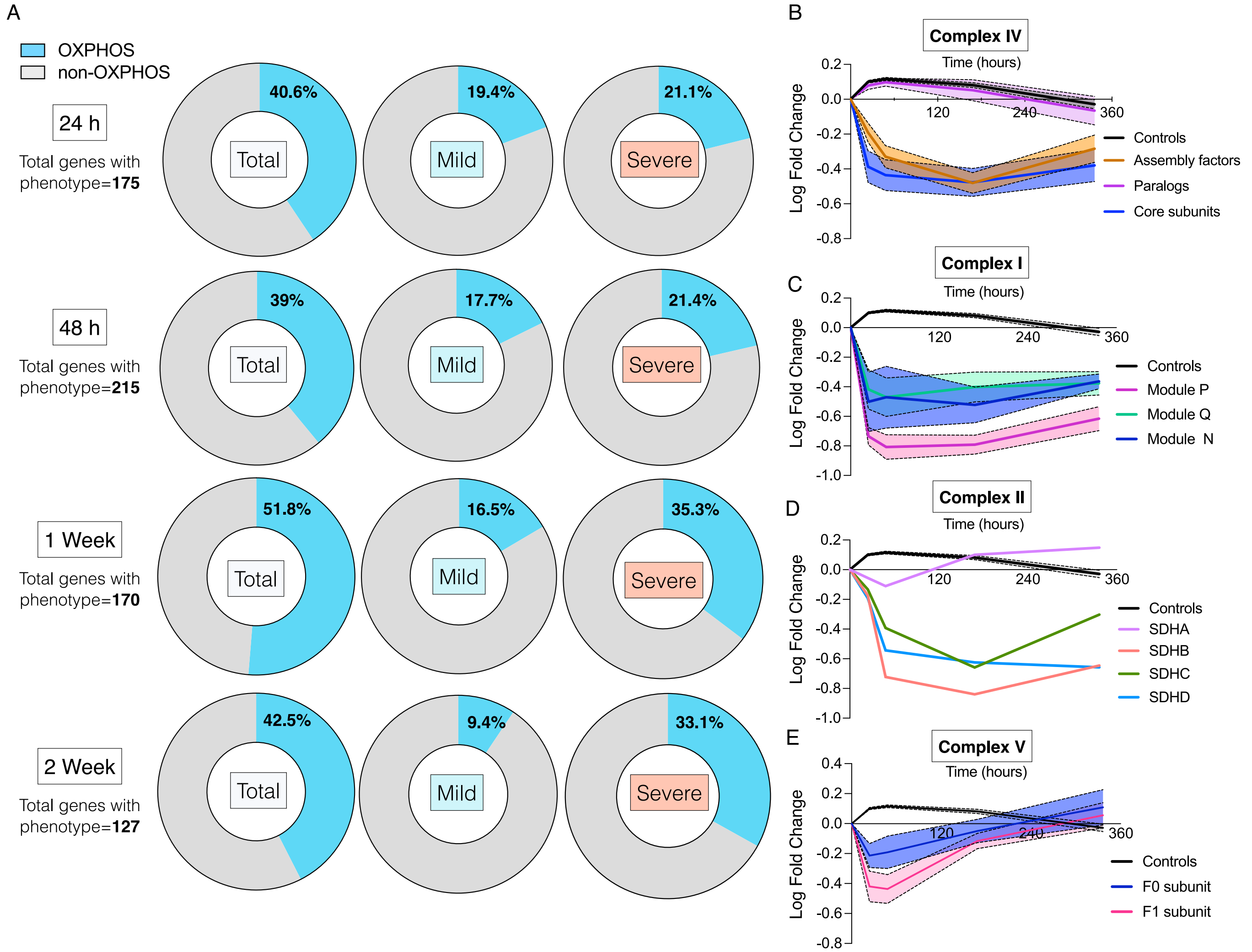


**Figure EV3. Phenotype of OXPHOS genes in the screening.**

**(A)** Donut charts showing the percentage of OXPHOS versus non-OXPHOS-related genes that scored positive in the screening. The charts illustrate the total number of genes identified, along with those exhibiting both mild and severe phenotypes across various time points. A drop in the fold change in the gal/glu ratio is considered a positive phenotype when it exceeds 20%, with a mild phenotype between 20 and 35%, and a severe phenotype above 35%. **(B–E)** Temporal dynamic analysis of the logarithmic fold change in the gal/glu ratio of genes involved in the assembly or function of Complexes IV **(B)**, I **(C)**, II **(D)**, and V **(E)** of the OXPHOS system. Source data are available online for this figure.

 © The Author(s)

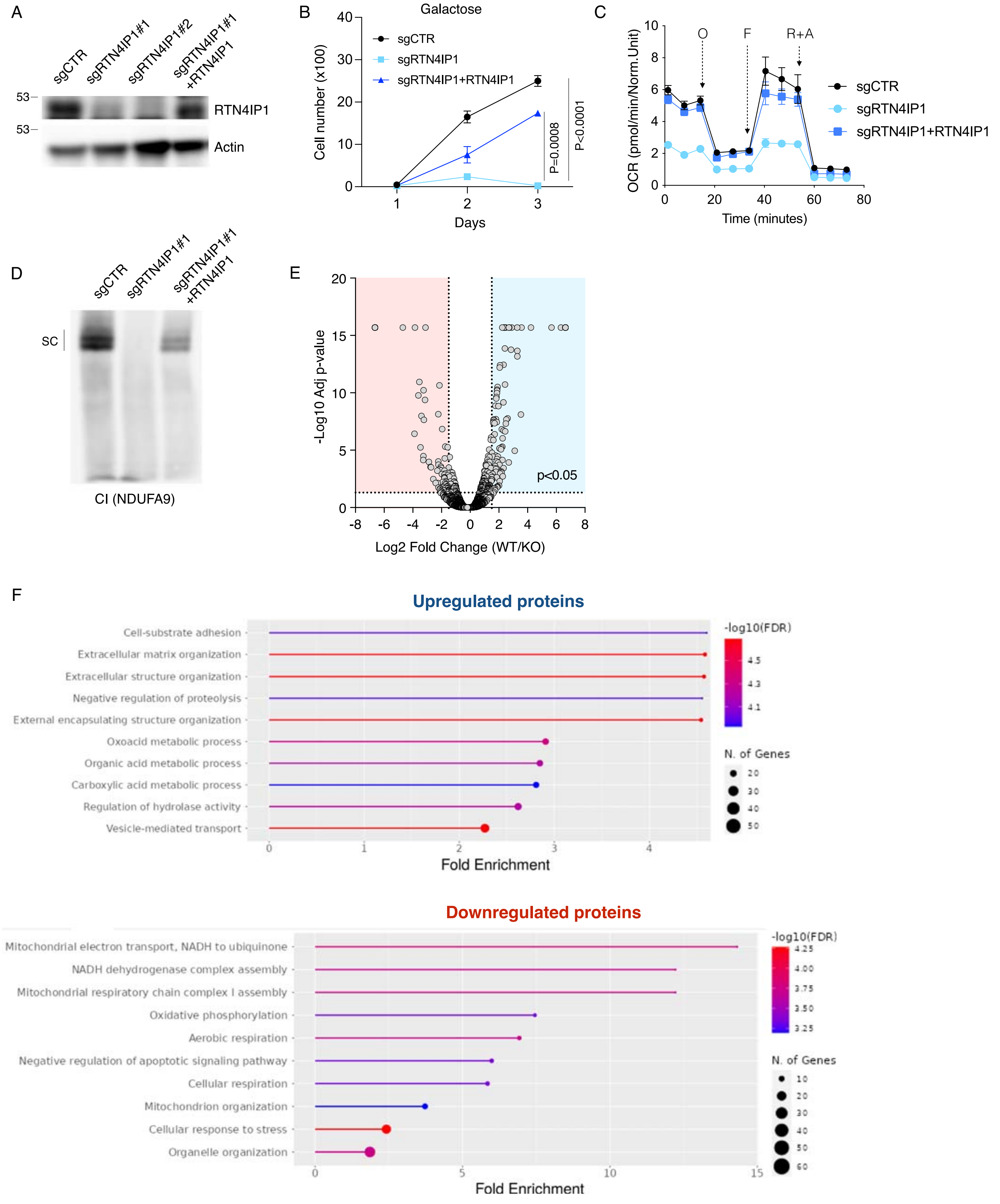
A
sgCTR
sgRTN4IP1#1
sgRTN4IP1#2
sgRTN4IP1#1 +RTN4IP1
53
RTN4IP1
53
Actin
B
Galactose
sgCTR
sgRTN4IP1
sgRTN4IP1+RTN4IP1
Cell number (x100)
Days
P=0.0008
P<0.0001
C
O
F
R+A
OCR (pmol/min/Norm.Unit)
Time (minutes)
sgCTR
sgRTN4IP1
sgRTN4IP1+RTN4IP1
D
sgCTR
sgRTN4IP1#1
sgRTN4IP1#1 +RTN4IP1
SC
CI (NDUFA9)
E
-Log10 Adj p-value
p<0.05
Log2 Fold Change (WT/KO)
F
Upregulated proteins
Cell-substrate adhesion
Extracellular matrix organization
Extracellular structure organization
Negative regulation of proteolysis
External encapsulating structure organization
Oxoacid metabolic process
Organic acid metabolic process
Carboxylic acid metabolic process
Regulation of hydrolase activity
Vesicle-mediated transport
Fold Enrichment
-log10(FDR)
N. of Genes
Downregulated proteins
Mitochondrial electron transport, NADH to ubiquinone
NADH dehydrogenase complex assembly
Mitochondrial respiratory chain complex I assembly
Oxidative phosphorylation
Aerobic respiration
Negative regulation of apoptotic signaling pathway
Cellular respiration
Mitochondrion organization
Cellular response to stress
Organelle organization
Fold Enrichment
-log10(FDR)
N. of Genes

**Figure EV4. Functional rescue and proteomic profiling of RTN4IP1 KO cells.**

(**A**) Immunodetection of RTN4IP1 in control cells, two stable RTN4IP1 KO clones, and KO cells with ectopic RTN4IP1 expression for rescue. Actin served as a loading control. (**B–D**) Rescue experiments showing that ectopic RTN4IP1 expression restores galactose-mediated cell survival (**B**), oxygen consumption (**C**), and Complex I levels (**D**) in RTN4IP1 KO cells. (**E**) Volcano plot illustrating the differential expression of proteins between wild-type (WT) and RTN4IP1 knockout (KO) HeLa cells. (**F**) Gene Ontology (GO) enrichment analysis showing enriched biological processes, cellular components, and molecular functions from this dataset. Immunoblots shown are representative of >3 independent experiments, and data were presented as means ± SEM. The statistical significance of the differences between groups was determined by a paired two-tailed Student's *t*-test (**B**, **E**). To account for multiple comparisons, *p* values were adjusted using the Benjamini–Hochberg false discovery rate (FDR) method. O oligomycin, F FCCP, R + A rotenone plus antimycin a. Source data are available online for this figure.

 © The Author(s)

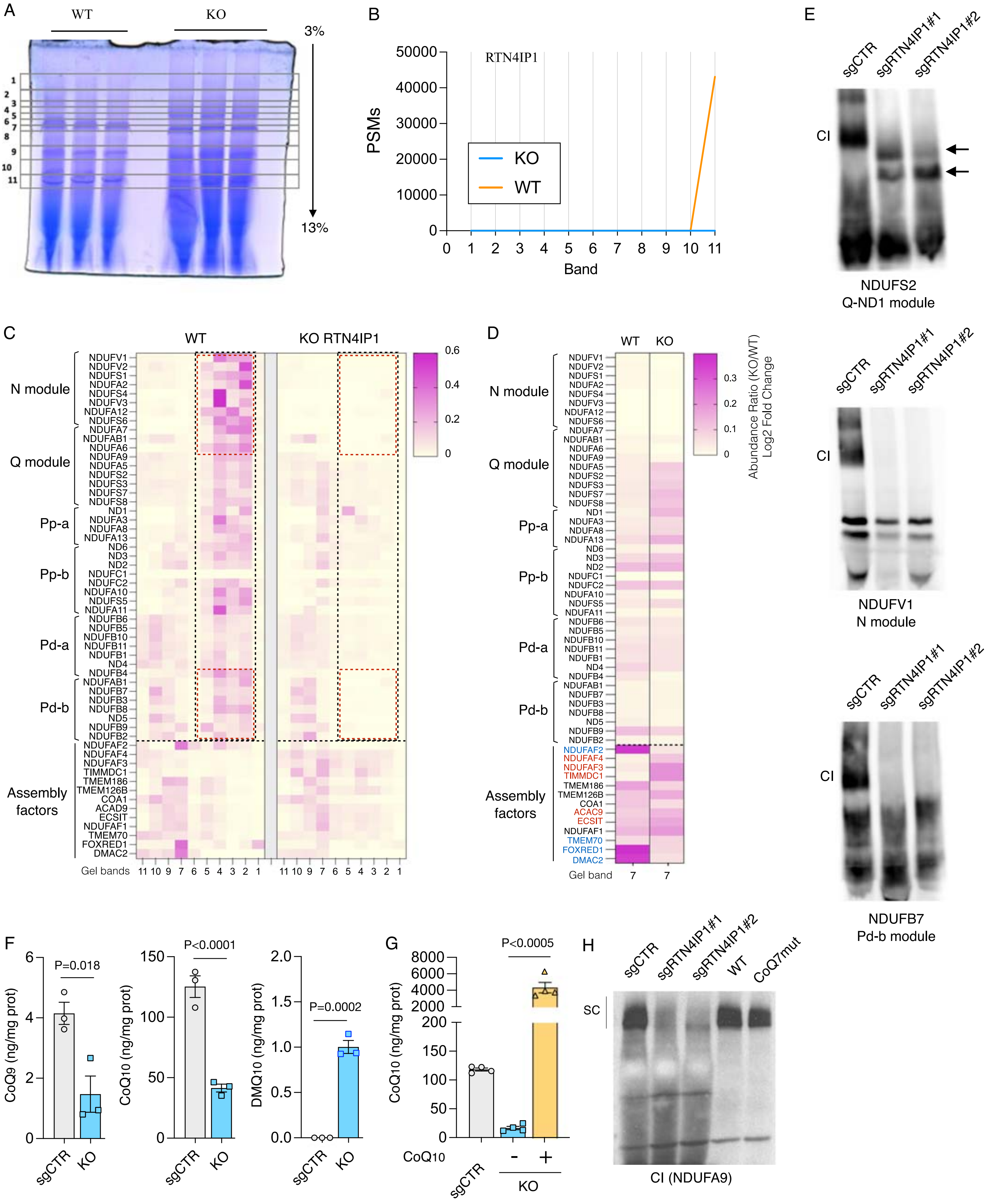
A
WT
KO
3%
13%
B
RTN4IP1
PSMs
KO
WT
Band
E
sgCTR
sgRTN4IP1#1
sgRTN4IP1#2
CI
NDUFS2
Q-ND1 module
NDUFV1
N module
NDUFB7
Pd-b module
C
WT
KO RTN4IP1
N module
Q module
Pp-a
Pp-b
Pd-a
Pd-b
Assembly factors
Gel bands
D
WT
KO
Abundance Ratio (KO/WT)
Log2 Fold Change
Gel band
F
CoQ9 (ng/mg prot)
P=0.018
CoQ10 (ng/mg prot)
P<0.0001
DMQ10 (ng/mg prot)
P=0.0002
sgCTR
KO
G
CoQ10 (ng/mg prot)
P<0.0005
CoQ10
H
sgCTR
sgRTN4IP1#1
sgRTN4IP1#2
WT
CoQ7mut
SC
CI (NDUFA9)

**Figure EV5. Comprehensive BN-PAGE and proteomic profiling reveal Complex I assembly impairments and CoQ dysregulation in RTN4IP1-deficient cells.**

(**A**) BN-PAGE of isolated mitochondria from WT RTN4IP1 and KO HeLa cells depicting the eleven bands that were sliced and subjected to proteomic analysis. (**B**) Peptides corresponding to the RTN4IP1 protein were identified in the previous samples, demonstrating their reduced abundance in the KO samples. (**C**) Heatmap showing differences in complex I subunits and assembly factors between WT and RTN4IP1 KO HeLa cells. The most notable differences, corresponding to mature complex I and CI-containing supercomplexes, are highlighted with dashed lines. (**D**) Heatmap focused on band 7, which showed the most striking differences in the accumulation and decrease of several assembly factors. (**E**) BN-PAGE analysis of DDM-solubilized mitochondria assessing multiple CI modules in WT and RTN4IP1 KO HeLa cells. The Q-ND1 module was identified using NDUFS2, the N-module using NDUFV1, and the Pd-b module using NDUFB7. Arrows indicate accumulated submodules containing NDUFS2 specifically in RTN4IP1 KO cells. (**F**) Relative level of CoQ9, CoQ10, and DMQ10 in control and RTN4IP1 KO HeLa cells. (**G**) Restoration of CoQ10 levels in RTN4IP1 KO HeLa cells after supplementing these cells with CoQ10 (20uM) for 1 week. (**H**) Blue native PAGE (BN-PAGE) showing the levels of CI in control and two stable clones of RTN4IP1 KO HeLa cells, along with healthy human skin fibroblasts (WT) and those carrying a pathogenic variant in the COQ7 gene. Immunoblots shown are representative of >3 independent experiments, and data were presented as means ± SEM. The statistical significance of the differences between groups was determined by a paired two-tailed Student's *t*-test (**F**) and one-way ANOVA (**G**). PSMs peptide-spectrum matches. Source data are available online for this figure.

 © The Author(s)

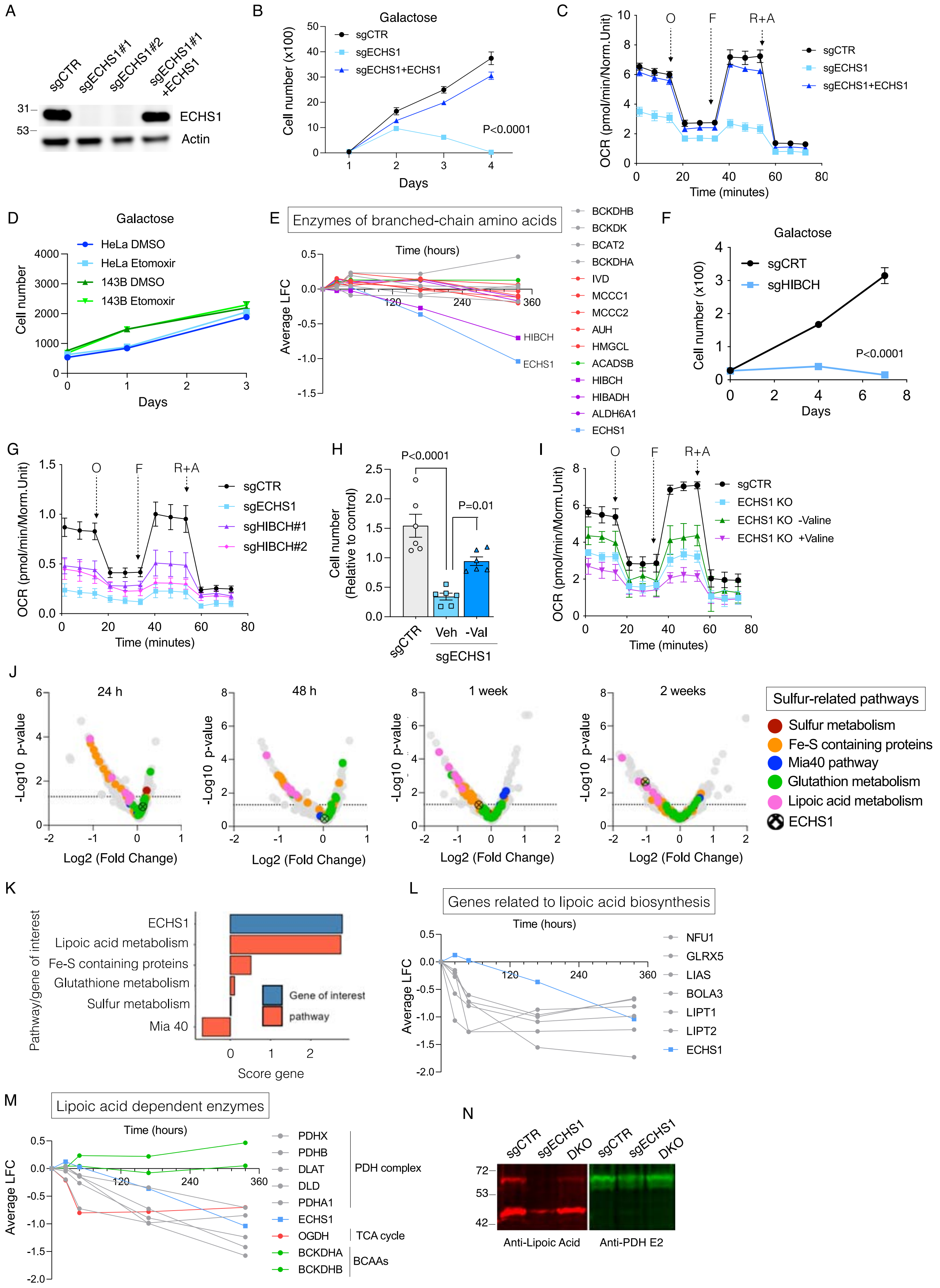
A
sgCTR
sgECHS1#1
sgECHS1#2
sgECHS1#1 +ECHS1
31
53
ECHS1
Actin
B
Galactose
sgCTR
sgECHS1
sgECHS1+ECHS1
Cell number (x100)
P<0.0001
Days
C
O
F
R+A
OCR (pmol/min/Norm.Unit)
Time (minutes)
D
Galactose
HeLa DMSO
HeLa Etomoxir
143B DMSO
143B Etomoxir
Cell number
Days
E
Enzymes of branched-chain amino acids
Time (hours)
Average LFC
HIBCH
ECHS1
BCKDHB
BCKDK
BCAT2
BCKDHA
IVD
MCCC1
MCCC2
AUH
HMGCL
ACADSB
HIBCH
HIBADH
ALDH6A1
ECHS1
F
Galactose
sgCRT
sgHIBCH
Cell number (x100)
P<0.0001
Days
G
OCR (pmol/min/Morm.Unit)
sgCTR
sgECHS1
sgHIBCH#1
sgHIBCH#2
Time (minutes)
H
P<0.0001
P=0.01
Cell number (Relative to control)
sgCTR
Veh
-Val
sgECHS1
I
OCR (pmol/min/Morm.Unit)
sgCTR
ECHS1 KO
ECHS1 KO -Valine
ECHS1 KO +Valine
Time (minutes)
J
24 h
48 h
1 week
2 weeks
-Log10 p-value
Log2 (Fold Change)
Sulfur-related pathways
Sulfur metabolism
Fe-S containing proteins
Mia40 pathway
Glutathion metabolism
Lipoic acid metabolism
ECHS1
K
ECHS1
Lipoic acid metabolism
Fe-S containing proteins
Glutathione metabolism
Sulfur metabolism
Mia 40
Gene of interest
pathway
Pathway/gene of interest
Score gene
L
Genes related to lipoic acid biosynthesis
Time (hours)
Average LFC
NFU1
GLRX5
LIAS
BOLA3
LIPT1
LIPT2
ECHS1
M
Lipoic acid dependent enzymes
Time (hours)
Average LFC
PDHX
PDHB
DLAT
DLD
PDHA1
ECHS1
OGDH
BCKDHA
BCKDHB
PDH complex
TCA cycle
BCAAs
N
sgCTR
sgECHS1
DKO
72
53
42
Anti-Lipoic Acid
Anti-PDH E2

**Figure EV6. ECHS1 deficiency impairs valine catabolism and lipoic acid–dependent enzyme function.**

(**A**) Immunodetection of ECHS1 in control cells, two stable ECHS1 KO clones, and KO cells with ectopic ECHS1 expression for rescue. Actin served as a loading control. (**B**, **C**) Rescue experiments showing that ectopic ECHS1 expression restores galactose-mediated cell survival (**B**) and oxygen consumption (**C**) in ECHS1 KO cells. (**D**) Growth curves depicting the proliferation of HeLa and 143B cells treated with either DMSO or etomoxir. (**E**) Evolution of the death phenotype (logarithmic fold change in the gal/glu ratio) of all the enzymes involved in the metabolism of branched-chain amino acids in the screening. (**F**) Cell number under galactose conditions in control and HIBCH KO HeLa cells. (**G**) Seahorse analysis comparing control, HIBCH KO and ECHS1 KO HeLa cells. (**H**) Cell number under galactose conditions at 72 h in control HeLa cells and ECHS1 KO HeLa cells in medium with (Veh) and without valine (-Val). (**I**) Seahorse analysis comparing control and ECHS1 KO cells cultured under conditions without valine and with valine supplementation (4 mM). (**J**) Volcano plots highlighting the scored values (logarithmic fold change in the gal/glu ratio) of genes involved in sulfur-related pathways and ECHS1 at various time points. (**K**) Manually curated enrichment analysis of mitochondrial pathways involving thiol groups at two weeks reveals that the ECHS1 score aligns specifically with the lipoic acid pathway. Scores were calculated as logFC * -logPvalue, reflecting the impact of gene knockouts on OXPHOS. The graph depicts the average pathway scores alongside ECHS1. (**L**, **M**) Temporal evolution of the death phenotypes (logarithmic fold change in the gal/glut ratio) associated with genes involved in lipoic acid biogenesis (**L**) and enzymes utilizing lipoic acid as a cofactor (**M**) and ECHS1. (**N**) Immunodetection of lipoylated proteins (red color) and the E2 subunit of the PDH complex (green) in mitochondrial extracts of control, ECHS1 KO, and double KO cells for both ECHS1 and ACAD8. Note that the lipoylated signal from PDH E2 is reduced in ECHS1 KO cells. Antibodies were incubated simultaneously on the same membrane and detected with different secondary fluorescent antibodies. Immunoblots shown are representative of >3 independent experiments, and data were presented as means ± SEM. Growth curves are calculated from three replicates and OCR is calculated from five replicates. The statistical significance of the differences between groups was determined by a paired two-tailed Student's *t*-test (**B**, **F**) and one-way ANOVA (**H**). O oligomycin, F FCCP, R + A rotenone plus antimycin a. Source data are available online for this figure.

 © The Author(s)
